# Phospholipase C-Related Catalytically Inactive Protein (PRIP) Regulates Lipolysis in Adipose Tissue by Modulating the Phosphorylation of Hormone-Sensitive Lipase

**DOI:** 10.1371/journal.pone.0100559

**Published:** 2014-06-19

**Authors:** Toshiya Okumura, Kae Harada, Kana Oue, Jun Zhang, Satoshi Asano, Masaki Hayashiuchi, Akiko Mizokami, Hiroto Tanaka, Masahiro Irifune, Nobuyuki Kamata, Masato Hirata, Takashi Kanematsu

**Affiliations:** 1 Department of Cellular and Molecular Pharmacology, Institute of Biomedical and Health Sciences, Hiroshima University, Hiroshima, Japan; 2 Department of Oral Surgery, Institute of Biomedical and Health Sciences, Hiroshima University, Hiroshima, Japan; 3 Department of Dental Anesthesiology, Institute of Biomedical and Health Sciences, Hiroshima University, Hiroshima, Japan; 4 Laboratory of Molecular and Cellular Biochemistry, Faculty of Dental Science, Kyushu University, Fukuoka, Japan; University of Graz, Austria

## Abstract

Phosphorylation of hormone-sensitive lipase (HSL) and perilipin by protein kinase A (PKA) promotes the hydrolysis of lipids in adipocytes. Although activation of lipolysis by PKA has been well studied, inactivation *via* protein phosphatases is poorly understood. Here, we investigated whether phospholipase C-related catalytically inactive protein (PRIP), a binding partner for protein phosphatase 1 and protein phosphatase 2A (PP2A), is involved in lipolysis by regulating phosphatase activity. *PRIP* knockout (*PRIP*-KO) mice displayed reduced body-fat mass as compared with wild-type mice fed with standard chow *ad libitum*. Most other organs appeared normal, suggesting that mutant mice had aberrant fat metabolism in adipocytes. HSL in *PRIP*-KO adipose tissue was highly phosphorylated compared to that in wild-type mice. Starvation of wild-type mice or stimulation of adipose tissue explants with the catabolic hormone, adrenaline, translocated both PRIP and PP2A from the cytosol to lipid droplets, but the translocation of PP2A was significantly reduced in *PRIP*-KO adipocytes. Consistently, the phosphatase activity associated with lipid droplet fraction in *PRIP*-KO adipocytes was significantly reduced and was independent of adrenaline stimulation. Lipolysis activity, as assessed by measurement of non-esterified fatty acids and glycerol, was higher in *PRIP*-KO adipocytes. When wild-type adipocytes were treated with a phosphatase inhibitor, they showed a high lipolysis activity at the similar level to *PRIP*-KO adipocytes. Collectively, these results suggest that PRIP promotes the translocation of phosphatases to lipid droplets to trigger the dephosphorylation of HSL and perilipin A, thus reducing PKA-mediated lipolysis.

## Introduction

Obesity is associated with the development of type 2 diabetes mellitus, coronary heart disease, an increased incidence of cancer, and respiratory complications (*e.g.*, obstructive sleep apnea) [1]. Obesity is characterized by excessive body fat accumulation in adipose tissue. Excess energy is stored as triacylglycerol (TAG) in lipid droplets, while lipolysis of stored TAG followed by the release of both non-esterified fatty acids (NEFA) and glycerol is stimulated by food deprivation (fasting) or stress. Ultimately, NEFA and glycerol are released from the adipocytes and transported to other tissues by circulation [2], [3]. Stepwise breakdown of TAG is catalyzed by a series of enzymes such as adipocyte triglyceride lipase (ATGL), hormone-sensitive lipase (HSL), and monoacylglycerol lipase (MGL) that produce diacylglycerol and monoacylglycerol as intermediates and eventually liberate three fatty acids and a glycerol [2], [3]. Lipolysis is regulated by several molecules, including adenosine 3′,5′-cyclic monophosphate (cAMP)-dependent protein kinase A (PKA) [4], [5], cAMP phoshodiesterase 3 or 4 [6] protein phosphatase 1 (PP1) [7], protein phosphatase 2A (PP2A) [8], [9], and perilipin A, a lipid droplet-associated protein [10], [11].

Catabolic hormones (*e.g.*, adrenaline and glucagon) trigger a signaling cascade that induces PKA activation. PKA then phosphorylates downstream targets, including HSL [4] and perilipin A [12] in adipocytes. The functions of HSL are controlled by multiple phosphorylation events: HSL is phosphorylated by PKA at Ser660, triggering its translocation to lipid droplets; it then forms a stable association with lipid droplets [13]. The subsequent phosphorylation of HSL Ser563 on the surface of the lipid droplets promotes the intrinsic catalytic activity of the lipase [14]. In turn, these residues can be dephosphorylated by PP2A, which inactivates the lipolytic activity [8], [9]. Adipocytes contain both PP1 and PP2A [8], but the precise mechanisms regarding the involvement of these phosphatases in regulating lipolysis have not been elucidated.

Phospholipase C-related catalytically inactive protein (PRIP) was initially identified as an inositol 1,4,5-trisphosphate-binding protein and later characterized as a protein with a domain organization similar to that of phospholipase C-δ but with no enzymatic activity. PRIP has two isoforms: PRIP1 and PRIP2 [15]–[20]. The functional aspects of PRIP have been elucidated by analyzing *PRIP1* knockout (*PRIP1*-KO) mice and *PRIP1* and *PRIP2* double-knockout (*PRIP*-DKO) mice [21], as well as by studying PIRP binding partners including GABA_A_ receptor-associated protein (GABARAP) [22], GABA_A_ receptor β subunit [23], phosphorylated Akt [24], PP1 and PP2A [25]–[27], and the syntaxin/SNAP-25 complex [28], [29]. PRIP facilitates GABARAP-mediated cell surface expression of γ2 subunit-containing GABA_A_ receptors by promoting the interaction between GABARAP and γ2 subunit of the receptors [22], [30], [31]. PRIP also regulates the phosphorylation level of the GABA_A_ receptor β subunit by binding with PP1 and PP2A [23], [26]. In addition, PRIP modulates GABA_A_ receptor trafficking *via* its association with PP1, PP2A and phosphorylated Akt [24], [26], and regulates dense-core vesicle exocytosis [28], [29].

In this study, we investigated the molecular mechanisms underlying reduced fat mass observed in *PRIP*-DKO mice and found that lipolytic activity in the adipocytes of the mutant mice was up-regulated. Importantly, our results show that PRIP, together with PP2A, regulates lipolysis by controlling phosphorylation-dependent HSL lipolytic activity.

## Materials and Methods

### Animals and ethics statement

*PRIP1*-KO and *PRIP2*-KO mice were generated as described previously [22], [32] and were backcrossed against the C57BL/6J strain for at least 7 and 2 generations, respectively. A *PRIP*-DKO mouse strain and its corresponding wild-type (WT) strain were generated by crossing the *PRIP1*-KO mice with the *PRIP2*-KO mice [26], [30]. The mice were reared in a pathogen-free facility at Hiroshima University, Japan with a 12 h light/dark cycle and at 23°C±2°C. Standard laboratory chow (MF; Oriental Yeast Co. Ltd., Tokyo, Japan) and water were available *ad libitum*. All experiments used male mice at 10–14 weeks of age except for the weight measurement of whole body and several tissues. This study was approved by the Animal Care and Use Committee of Hiroshima University (Permission Numbers: A11–12, A11–56, A12–98) and performed in accordance with the Guide for Hiroshima University Animal Experimentation Regulation.

### Histochemistry

Epididymal white adipose tissue was fixed in 10% formalin/phosphate-buffered saline (PBS) and then embedded in paraffin. Sections were subjected to standard hematoxylin-eosin staining. Cell area was measured using ImageJ software, a freeware image analysis program developed at the National Institutes of Health.

### Measurement of plasma leptin and adiponectin

Blood samples were obtained from the heart, and plasma leptin and adiponectin levels were measured using a mouse leptin ELISA kit (Morinaga Institute of Biological Science, Yokohama, Japan) and a mouse adiponectin ELISA kit (BioVendor, Modrice, Czech Republic) according to the manufacturers' instructions.

### Analysis of lipid metabolism

NEFA and glycerol levels in blood or culture medium were determined enzymatically using Wako NEFA C test kit (Wako Pure Chemical Industries, Osaka, Japan) and a free glycerol assay kit (BioVision, Milpitas, CA), respectively, according to the manufacturers' instructions.

### Extraction and subcellular fractionation of epididymal white adipose tissue

Adipocyte subcellular fractionation was performed as previously described [33]. Briefly, epididymal white adipose tissue was homogenized in an ice-cold homogenization buffer (20 mM Tris-HCl at pH 7.2, 0.25 M sucrose, 50 mM sodium fluoride, 10 mM sodium pyrophosphate, 1 mM sodium orthovanadate, 2 mM EDTA, and 2 mM EGTA) containing protease inhibitors (1 mM phenylmethylsulphonyl fluoride, 100 µM (p-amidinophenyl) methanesulfonyl fluoride hydrochloride, 10 µg/mL leupeptin, 10 µg/mL pepstatin A, and 3.4 µg/mL aprotinin). The homogenate was centrifuged at 40,000×*g* for 20 min at 4°C. The resulting floating fat-cake fraction, infranatant fraction, and pellet fraction were collected. The infranatant fraction was centrifuged again to obtain the supernatant fraction. The content of HSL in subcellular fractions of the floating fat-cake and the supernatant was assessed by immunoblotting.

### Immunoblot analysis

The following primary antibodies were used: anti-β3 adrenergic receptor (ab59685) and anti-comparative gene identification 58 (CGI-58, also known as abhydrolase domain-containing protein 5 [Abhd5], ab58283) antibodies from Abcam (Cambridge, UK); anti-β-actin antibody (IMG-5142A) from Imgenex (San Diego, CA); anti-MGL antibody (LS-C138957) from Lifespan Biosciences (Seattle, WA); anti-GAPDH antibody (PA1-16781) from Thermo Fisher Scientific Inc. (Rockford, IL); anti-ATGL (#2138), anti-HSL (#4107), anti-p-HSL Ser660 (#4126), anti-p-HSL Ser563 (#4139), anti-perilipin (#9349), and anti-PP1 (#2582) antibodies from Cell Signaling Technology, Inc. (Danvers, MA); anti-PP2A antibody (#610555) from Becton, Dickinson and Co. (Franklin Lakes, NJ); and anti-p-perilipin Ser497 antibody (#4855; Ser497 of human perilipin 1A is equivalent to Ser492 in murine perilipin 1A) from Vala Sciences (San Diego, CA). Production of the anti-PRIP1 and anti-PRIP2 antibodies was described previously [22], [32]. Blocking One-P solution (Nacalai Tesque Inc., Kyoto, Japan) was used for phosphoprotein detection with specific primary phosphoantibodies. After overnight incubation at 4°C with the appropriate primary antibody, the immunoblot membrane was incubated with a horseradish peroxidase-conjugated secondary antibody (GE Healthcare Life Sciences, Uppsala, Sweden) and developed using a chemiluminescent substrate (SuperSignal West Femto, Thermo Fisher Scientific Inc.). The resulting signal was captured using an ImageQuant LAS 4000 mini system (GE Healthcare Japan Corporation, Tokyo, Japan). Densitometry values were calculated using the ImageJ software (National Institutes of Health).

### Preparation of cultured adipose tissues and related experiments

Organ culture was performed according to procedures described in previous studies [34], [35]. Briefly, freshly isolated epididymal fat pads (200–350 mg) were washed in PBS, minced into 5–10 mg pieces with scissors, and incubated in serum-free M199 (M7528, Sigma-Aldrich Co., St. Louis, MO) containing 25 mM HEPES and Earle's salts, as previously described [34], [35]. The tissue was kept in a 5% CO_2_ incubator at 37°C to equilibrate overnight. Pieces that were approximately 30–50 mg were then used in the subsequent experiments. The cultured adipose pieces were washed with the assay medium (the abovementioned serum-free M199 medium) and then incubated for 30 min with 1 µM adrenaline. Pieces were then homogenized in ice-cold lysis buffer at 4°C and fractionated into the floating fat-cake and supernatant fractions as described above. For the experiments examining the effects of adrenaline in combination with okadaic acid (OA), OA (1 µM) was added 20 min before stimulation with 1 µM adrenaline.

### Measurement of phosphatase activity

Phosphatase activity was measured using the ProFluor Ser/Thr PPase assay kit (Promega, Madison, WI) as previously described [36], [37] with minor modifications. Briefly, explants were stimulated with 1 µM adrenaline for 30 min, and then homogenized in ice-cold homogenization buffer at 4°C and fractionated into the floating fat-cake and supernatant fractions as described above. The floating fat-cake fractions were lysed by adding Triton-X 100 (final concentration of 0.2%) and mixed with anti-PP2A or anti-PP1 antibodies and control IgG overnight at 4°C. The samples were further incubated with protein G-sepharose beads (GE Healthcare, Uppsala, Sweden) for 2 hours at 4°C. After washing with 0.2% Triton-X 100/Tris-buffered saline, the immunoprecipitates were obtained by centrifugation and resuspended in a phosphatase dilution solution of the ProFluor Ser/Thr PPase assay kit. The assay was then performed according to the manufacturers' protocol.

### Dephosphorylation assay

To measure the dephosphorylation of HSL, cultured explants were stimulated with 5 µM adrenaline for 30 min and then treated with 20 µM SR59230A hydrochloride (a β3-adrenergic receptor antagonist; Tocris Bioscience, R&D Systems, Shanghai, China) and 10 µM myristoylated PKI 14–22 amide (a PKA inhibitor; #2546; Tocris Bioscience). The explants were homogenized in a homogenization buffer, and the floating fat-cake fractions were obtained by centrifugation and were analyzed by western blotting using anti-p-HSL antibodies.

### Cell culture and immunocytochemistry

COS7 cells (JCRB9127) were obtained from the Japanese Collection of Research Bioresources (Health Science Research Resources Bank, Osaka, Japan) and cultured with 10% FBS-containing DMEM medium (Sigma). GFP-conjugated PRIP plasmid was transfected using Lipofectamine 2000 (Life Technologies, Carlsbad, CA), according to the manufacturer's protocols. Five hours after transfection, the medium was changed to DMEM containing 10% FBS and 400 µM oleic acid to induce the formation of lipid droplets. Cells were cultured overnight, stimulated with 5 µM adrenaline for 5 min, and then fixed with 4% formaldehyde and 0.05% TritonX-100 in PBS. PP2A and PP1 were stained with specific antibodies, and lipid droplets were visualized using HCS LipidTOX Red Neutral Lipid Stain (Molecular Probe, Life Technologies).

### Data analysis

Data are expressed as mean ±SEM. Statistical analysis was performed using the Student's *t*-test or ANOVA, followed by the contrast test with Tukey's error protection. Differences were considered significant at *P*<0.05.

## Results

### Impaired fat metabolism in *PRIP*-DKO mice

**Figure 1:**
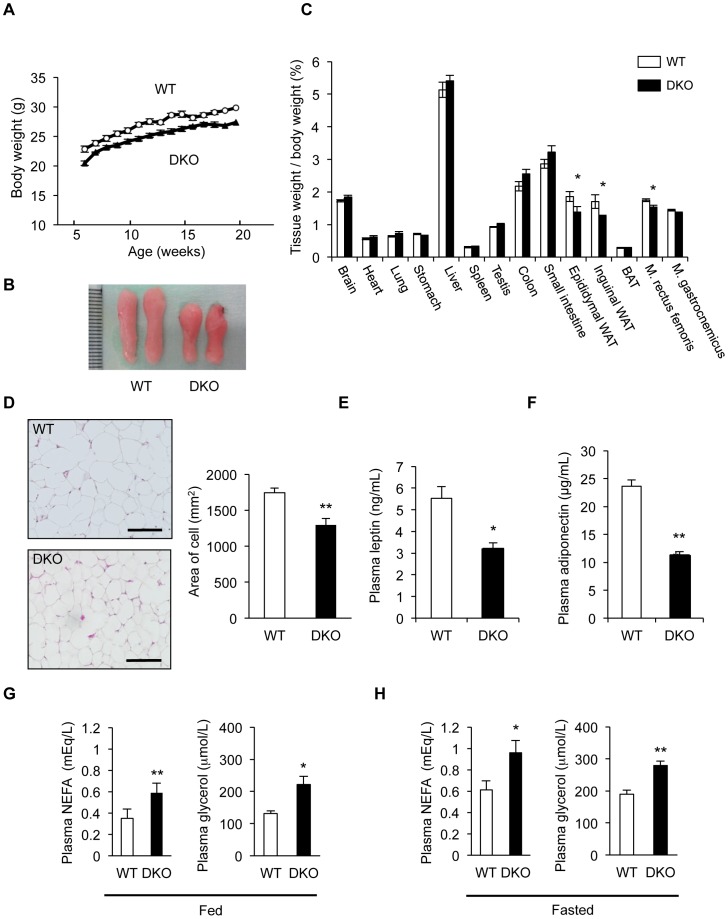
Impairment of fat metabolism in *PRIP*-DKO mice. (A) Body weight changes in WT (open circle) and *PRIP*-DKO (DKO, closed triangle) male mice monitored 20 weeks after birth (n = 5 mice for both genotypes). (B) Comparison of the epididymal fat pad of WT and *PRIP*-DKO mice at the age of 10-week-old. (C) Comparison of wet tissue weight (representing major organs) obtained from WT and *PRIP*-DKO male mice at the age of 18-week-old. The graph shows percent of tissue weight against body weight [26.6±0.8 g (WT), 23.4±0.6 g (*PRIP*-DKO); n = 5 mice per genotype]. WAT, white adipose tissue; BAT, brown adipose tissue; M. (Musculus) rectus femoris; M. (Musculus) gastrocnemius. (D) Comparison of adipocyte size. Graph shows average size of cells assessed using hematoxylin and eosin-stained sections (n = 3 sections for both genotypes). Scale bar: 100 µm. (E, F) Concentration of plasma leptin (n = 5 mice for both genotypes) and adiponectin (n = 7 mice for both genotypes) at the age of 10–12 weeks. Mice were fed a standard chow *ad libitum*. (G, H) Plasma NEFA and glycerol concentration at the age of 10–12 weeks under conditions of (G) *ad libitum* feeding (n = 3 mice for both genotypes) and (H) fasting for 8 h (n = 4 mice for both genotypes). The data represent mean ±SEM. *P<0.05 and **P<0.01 versus the corresponding WT value.

We found that *PRIP*-DKO mice fed with a standard chow *ad libitum* weighed less as monitored body weight from 6 to 20 weeks of age and had a smaller epididymal fat pad than WT mice (Fig. 1A, B), although the genotypes had similar body weights at 2 weeks of age (WT: 8.30±0.37 g, n = 10; *PRIP*-DKO: 8.08±0.19 g, n = 29; no statistical significance). We further measured weights of several other organs. As shown in Fig. 1C, the mean weights of epididymal and subcutaneous inguinal white adipose tissues to the body weight were smaller in *PRIP*-DKO mice by approximately 25% in both tissues compared to those in WT mice. The mean weight of musculus rectus femoris was also reduced in *PRIP*-DKO mice, but that of musculus gastrocnemius was similar in both genotypes. The other organs examined were similar in weight between two genotypes of mice. The anal-nasal body length (WT: 90.3±0.8 mm, n = 3; *PRIP*-DKO: 90.3±0.8 mm, n = 3) and tibia length (WT: 18.6±0.1 mm, n = 3; *PRIP*-DKO: 18.4±0.2 mm, n = 3) at 12 weeks of age were similar between the genotypes, suggesting that *PRIP*-DKO mice had normal growth.

Histological examination of hematoxylin-eosin-stained sections revealed that the average size of white adipocytes in *PRIP*-DKO mice was approximately 25% smaller than that in WT mice (Fig. 1D). Plasma levels of leptin and adiponectin in *PRIP*-DKO mice were lower than that in WT mice by approximately 50% (Fig. 1E, F), which likely corresponded to the smaller white adipose tissue. In addition, plasma levels of the TAG metabolites NEFA and glycerol were significantly increased under both *ad libitum* feeding and 8 h fasting (Fig. 1G, H). Since average food intake to the body weight during 10–12 weeks was similar between the genotypes at *ad libitum* feeding, nutritional status would also be similar in *PRIP*-DKO mice. Therefore, PRIP deficiency appeared to cause an impairment of fat metabolism by either decreased lipogenesis or increased lipolysis, or both.

### Increased HSL and perilipin phosphorylation in *PRIP*-DKO adipose tissue

**Figure 2:**
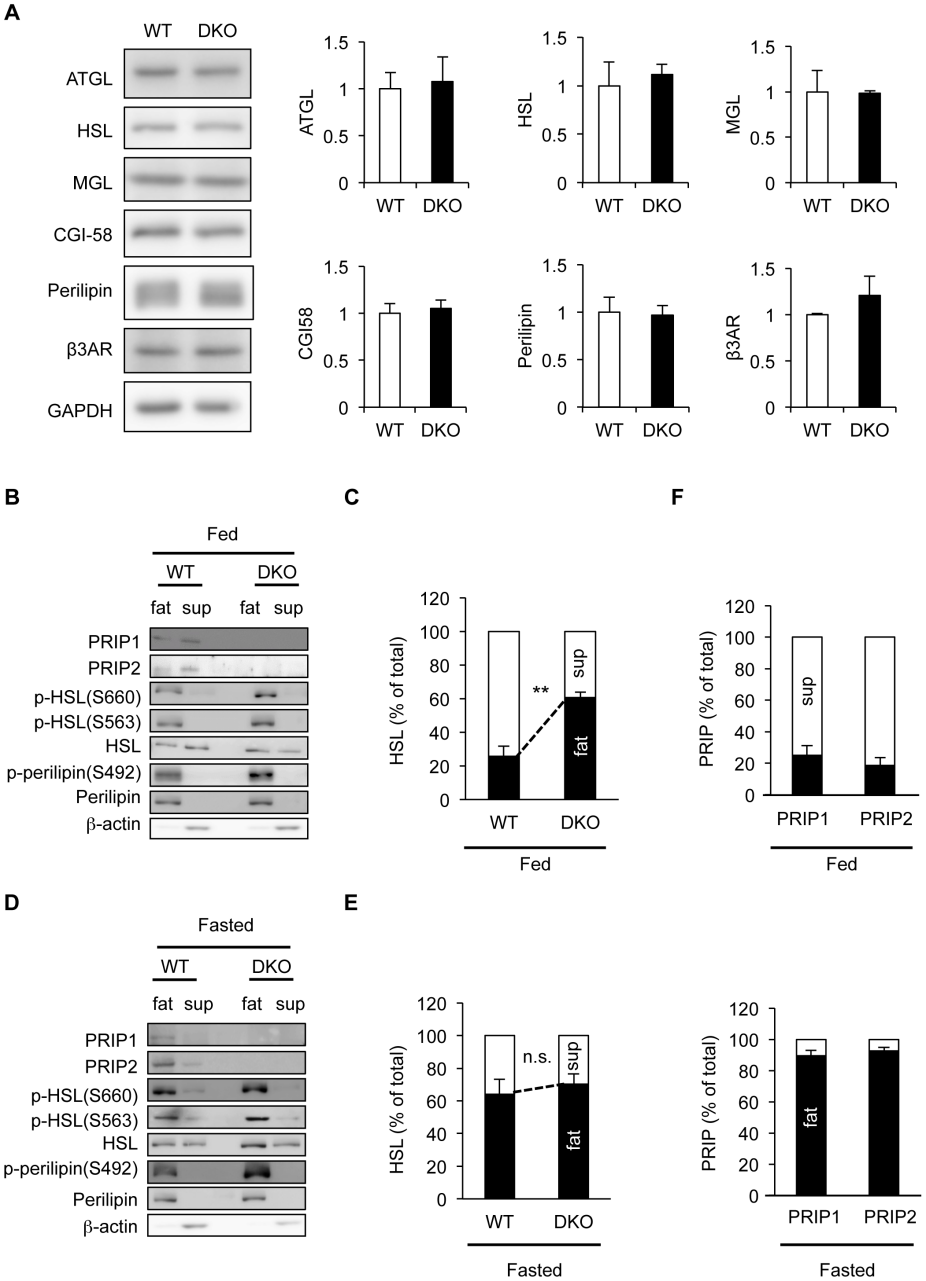
Increased phosphorylation of HSL and perilipin in *PRIP*-DKO adipocytes, and altered PRIP distribution after starvation. (A) Comparison of lipid metabolism-related proteins. Whole lysates obtained from WT and *PRIP*-DKO (DKO) epididymal fat pads were analyzed for western blotting using the indicated protein-specific antibodies. β3AR; β3-adrenergic receptor. The differences are not statistically significant. (B–F) Altered subcellular distribution of HSL, PRIP1, and PRIP2, and altered phosphorylation of HSL and perilipin in epididymal white adipose tissues prepared from WT and *PRIP*-DKO mice maintained under fed (B) and fasted (D) conditions. Whole lysates were fractionated by centrifugation into a floating fat-cake fraction, a supernatant fraction, and a pelleted membrane fraction. Western blotting was performed using the fat-cake (fat) and supernatant (sup) fractions. Indicated molecules were detected using specific antibody. Each image is a typical example from three experiments. Perilipin and β-actin are lipid droplet and cytosol marker proteins, respectively. Subcellular distribution of HSL under fed and fasted conditions is shown in (C) and (E), respectively. Subcellular distribution of PRIP1 and PRIP2 in fed (upper panel) and fasted (lower panel) conditions is shown in (F). The black and white bars represent the amount of HSL (C, E) and PRIP (F) in the fat and sup fractions, respectively. The amount of total HSL (C, E) and PRIP (F) in the fat and sup fractions is expressed as 100%. The data represent mean ±SEM. **P<0.01 and n.s. (not significant) versus the corresponding WT value.

To see if the lipolysis pathway was altered, we first examined the expression of various proteins involved in TAG metabolism, such as ATGL, HSL, MGL, CGI-58, perilipin A, and β3-adrenergic receptor in WT and *PRIP*-DKO white adipose tissues; an immunoblot analysis showed a similar level of these proteins between WT and mutant mice (Fig. 2A).

HSL, the rate-limiting enzyme for TAG hydrolysis, translocates from the cytosol to the surfaces of intracellular lipid droplets upon the onset of lipolysis [38], whose activity is regulated by reversible phosphorylation *via* PKA, PP1, and PP2A [4], [5], [7]–[9], [13], [14]. Perilipin A is a facilitator of TAG hydrolysis that also resides at the surface of lipid droplets and is phosphorylated by PKA [10], [11]. Therefore, we examined the subcellular localization of HSL and the phosphorylation levels of HSL and perilipin A in WT and *PRIP*-DKO white adipocytes. Homogenates of epididymal fat pads obtained from non-fasting and 8 h-fasting mice were fractionated by centrifugation into three fractions: a floating fat-cake fraction (lipid droplet fraction), a pelleted membrane fraction, and a supernatant fraction (cytosol). Immunoblotting with an anti-HSL antibody revealed that HSL was undetectable in the pelleted membrane fractions from both genotypes of mice (Fig. S1). Therefore, only the floating fat-cake and supernatant fractions were used for further experiments. In addition, perilipin A was only present in the fat-cake fractions in both genotypes (Fig. S1).

HSL in non-fasting WT mice was mainly observed in the cytosol (74.4%), while in *PRIP*-DKO mice it was mainly observed in the fat-cake fraction (60.5%; Fig. 2B, C). In WT mice fasted for 8 h, the proportion of HSL in the fat-cake fraction increased to 63.9%, which was similar to that in the fat-cake fraction from *PRIP*-DKO mice (70.1%; Fig. 2D, E). As the localization and activity of HSL are modulated by phosphorylation at Ser563 and Ser660 [13], the phospho-status of HSL was analyzed by the specific antibodies. As shown in Fig. 2B, the phosphorylation levels of Ser563 and Ser660 were higher in *PRIP*-DKO mice than in WT mice under fed condition. By contrast, fasting for 8 h elevated the phospho-levels similarly in two genotypes (Fig. 2D).

The amount of perilipin A detected in the floating fat-cake fraction was similar between WT and *PRIP*-DKO mice under fed and fasting conditions (Fig. 2B, D). It was reported that phosphorylation of perilipin A at Ser492 is required for maximal lipolysis and triggers a massive remodeling of lipid droplets that increases the surface area of lipid droplets available to lipases [39], [40]. Therefore, phosphorylation of perilipin A Ser492 was examined using a phosphospecific antibody; the results showed that *PRIP*-DKO mice exhibited higher phosphorylation of perilipin A Ser492 in adipose tissues under both fed and fasting conditions (Fig. 2B, D), indicating that more phosphorylated (active) perilipin A was present on lipid droplets in *PRIP*-DKO adipocytes. These results suggest that *PRIP*-DKO mice have higher lipolytic activity in adipose tissues, consistent with their reduction in fat-pad mass.

### Starvation induces PRIP translocation to lipid droplets in adipocytes

To elucidate the role of PRIP in lipolysis, we next examined subcellular distributions of PRIP1 and PRIP2 in epididymal adipose tissues from fed or fasting WT mice (see the WT floating fat-cake and supernatant lanes in Fig. 2B, D). Under fed condition, 74.9% and 81.6% of PRIP1 and PRIP2, respectively, were present in the cytosol (the upper panel in Fig. 2F), but the fasting drastically altered the localization; approximately 90% of both PRIP1 and PRIP2 was detected in fat-cake fractions (the lower panel in Fig. 2F), indicating that fasting triggers the translocation of PRIP to lipid droplet surfaces and thereby PRIP is involved in dephosphorylation of HSL and perilipin by bringing protein phosphatases.

### PRIP deficiency is linked to increased phosphorylation of HSL and perilipin in cultured adipose tissues

**Figure 3:**
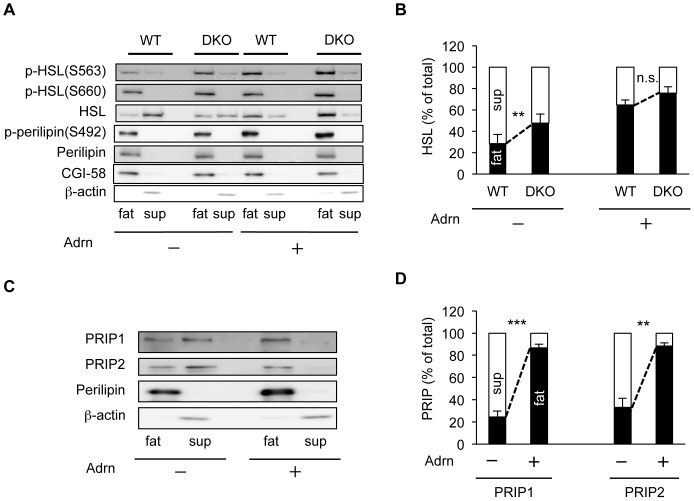
Alterations in subcellular PRIP distribution and HSL phosphorylation in explant adipose tissues. (A, B) Adrenaline stimulation (Adrn) induces HSL translocation from cytosol fraction (sup) to fat-cake fraction (fat) in WT and *PRIP*-DKO explant adipose tissues. The bars for fat (black) and sup (white) in B represent the floating fat-cake and supernatant fractions, respectively. The amount of total HSL in fat and sup fractions is expressed as 100%. All the data represent mean ±SEM. **p<0.01 and n.s. (not significant) versus the corresponding WT value. Altered phosphorylation of HSL at Ser563 and Ser660 and perilipin at Ser492 in adipose explant lipolysis assays with or without stimulation by 1 µM adrenaline are also shown in (A). A representative image is shown; similar images were obtained from three independent experiments. (C, D) PRIP translocation to the lipid droplet fraction in response to 1 µM adrenaline (Adrn) stimulation. The bars for fat (black) and sup (white) in (D) represent the floating fat-cake and supernatant fractions, respectively. The amount of total PRIP in fat and sup fractions is expressed as 100%. Perilipin and β-actin are lipid droplet and cytosol marker proteins, respectively. A representative image is shown; similar images were obtained from three independent experiments (C). The data represent mean ±SEM. **P<0.01 and ***P<0.001 versus the corresponding values of PRIP1 and PRIP2 without adrenaline stimulation.

To eliminate systemic influences from other organs, lipolysis assays were performed using explanted adipose tissues. Epididymal adipose tissue explants were cultured overnight and then stimulated with 1 µM adrenaline. Homogenates of the cultured explants were then separated into floating fat-cake, supernatant, and pellet fractions by centrifugation, and then the floating fat-cake and supernatant fractions were analyzed by western blotting. As shown in Fig. 3A and B, under basal conditions, the HSL content in the fat-cake fraction from *PRIP*-DKO mice was 1.7-fold higher than that from WT mice. However, when the explants were stimulated with adrenaline, the level of HSL was similar for WT and *PRIP*-DKO (approximately 63% and 71%, respectively). The HSL in the fat-cake fraction from basal *PRIP*-DKO explants was highly phosphorylated at Ser563 and Ser660 as compared with that from the corresponding WT explants, but the levels were similar with adrenaline stimulation (Fig. 3A). The amount of perilipin A in the floating fat-cake fraction was similar between WT and mutant mice, but, under basal conditions, the phosphorylation level of perilipin A was higher in *PRIP*-DKO mice than in WT mice (Fig. 3A). These results were consistent with those observed in the whole animal (Fig. 2 B–E). In addition, majority of CGI-58 was fractionated into the floating fat-cake fractions independent of genotypes and adrenaline stimulation (Fig. 3A).

### PRIP accumulates in lipid fractions in response to adrenaline stimulation

As shown in Fig. 3C and D, under basal conditions in explant analysis, PRIP1 and PRIP2 were mainly present in the cytosolic fraction (75.6 and 67.0%, respectively). However, after adrenaline stimulation, more than 80% of PRIP1 and PRIP2 were found in the fat-cake fraction, which is similar to the results seen in the whole-animal experiments (Fig. 2F).

### PRIP mediates translocation of protein phosphatases to the fat-cake fractions

**Figure 4:**
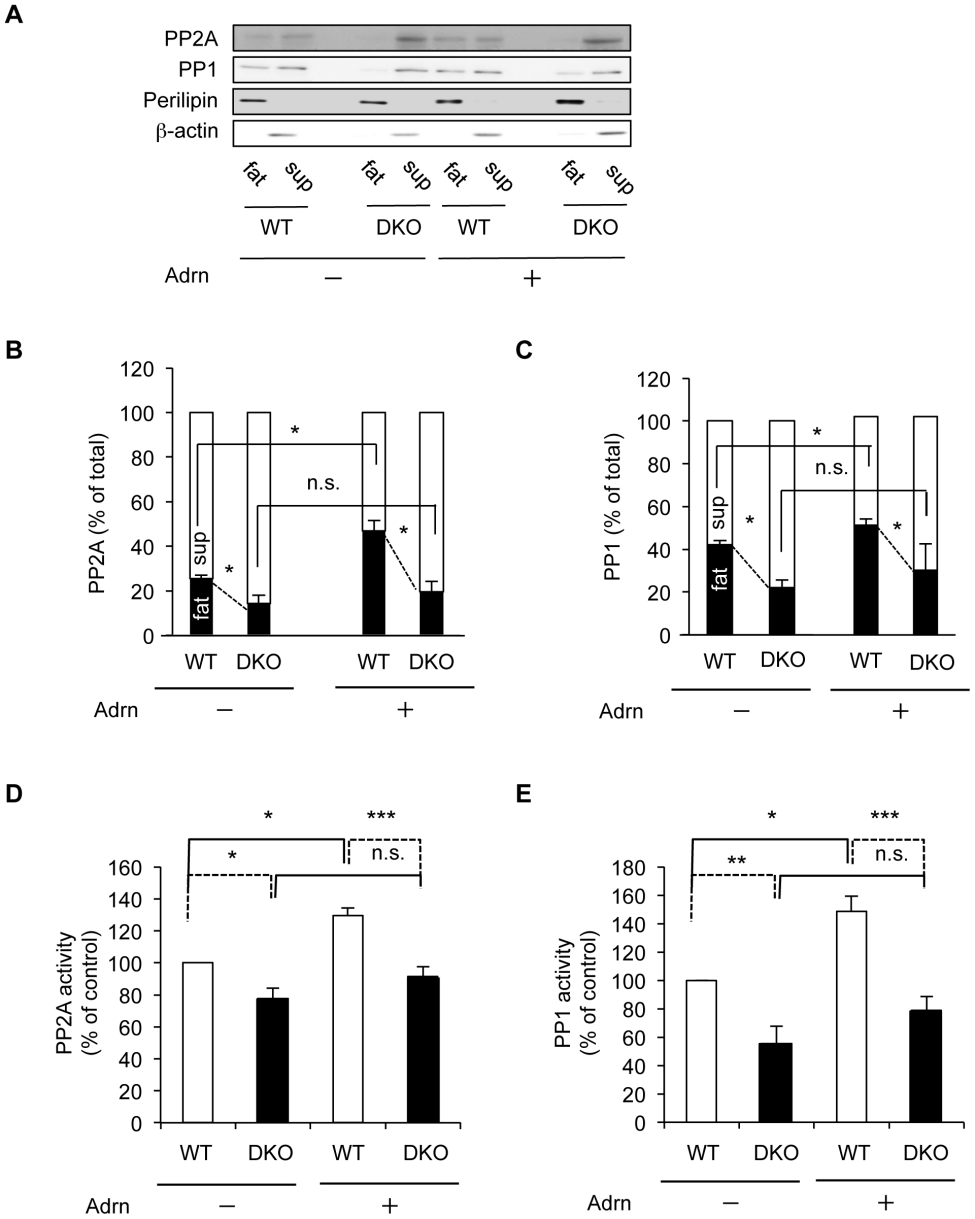
PP2A and PP1 are translocated from the cytosol to lipid droplets in adipocytes in response to adrenaline stimulation. (A–C) Translocation of PP2A (A, B) and PP1 (A, C) to the lipid droplet fraction in response to stimulation with 1 µM adrenaline (Adrn). The bar graph shows the amount of PP2A (B) and PP1 (C) in fat (black) and sup (white) fractions, respectively. A typical image from four independent experiments is shown (A). (D, E) Phosphatase activity in floating fat-cake fraction. Phosphatase activities of PP2A (D, n = 4) and PP1 (E, n = 5) were measured using floating fat-cake fractions from WT and *PRIP*-DKO explants treated with or without 1 µM adrenaline. The data represent mean ±SEM; *P<0.05, **P<0.01, ***P<0.001 and n.s. (not significant) versus the corresponding values for paired bars connected by solid and dotted lines (B–E).

To explore the molecular basis underlying PRIP deficiency-induced lipolytic changes in adipocytes, we next examined dephosphorylation of proteins involved in lipolysis in adipocytes. PRIP binds to the catalytic subunit of PP1 and PP2A [25]–[27], main phosphatases in the lipolysis pathway [2], [7]–[9]. Therefore, we examined the subcellular distributions of PP2A and PP1. The proportion of PP2A localized in the WT fat-cake fraction (25.5%) was higher by 1.8-fold than that in the *PRIP*-DKO (14.2%) under unstimulated conditions (Fig. 4A, B). When the explants were stimulated by adrenaline, the PP2A content in the fat-cake fraction was significantly increased to 46.9% in WT fat pads, however, the level of PP2A in *PRIP*-DKO mice did not change significantly. PP1 in adipocytes essentially behaved similarly to PP2A (Fig. 4A, C).

We further measured phosphatase activities in fat-cake fractions after the stimulation with adrenaline. Both PP2A and PP1 activities in *PRIP*-DKO adipocytes were lower than those of WT adipocytes. By adrenaline stimulation, the activities in fat-cake fraction significantly increased in WT adipocytes, whereas they did not differed in *PRIP*-DKO adipocytes (Fig. 4D and E).

### Adrenaline stimulation triggers the translocation of PRIP and PP2A to lipid droplets

**Figure 5:**
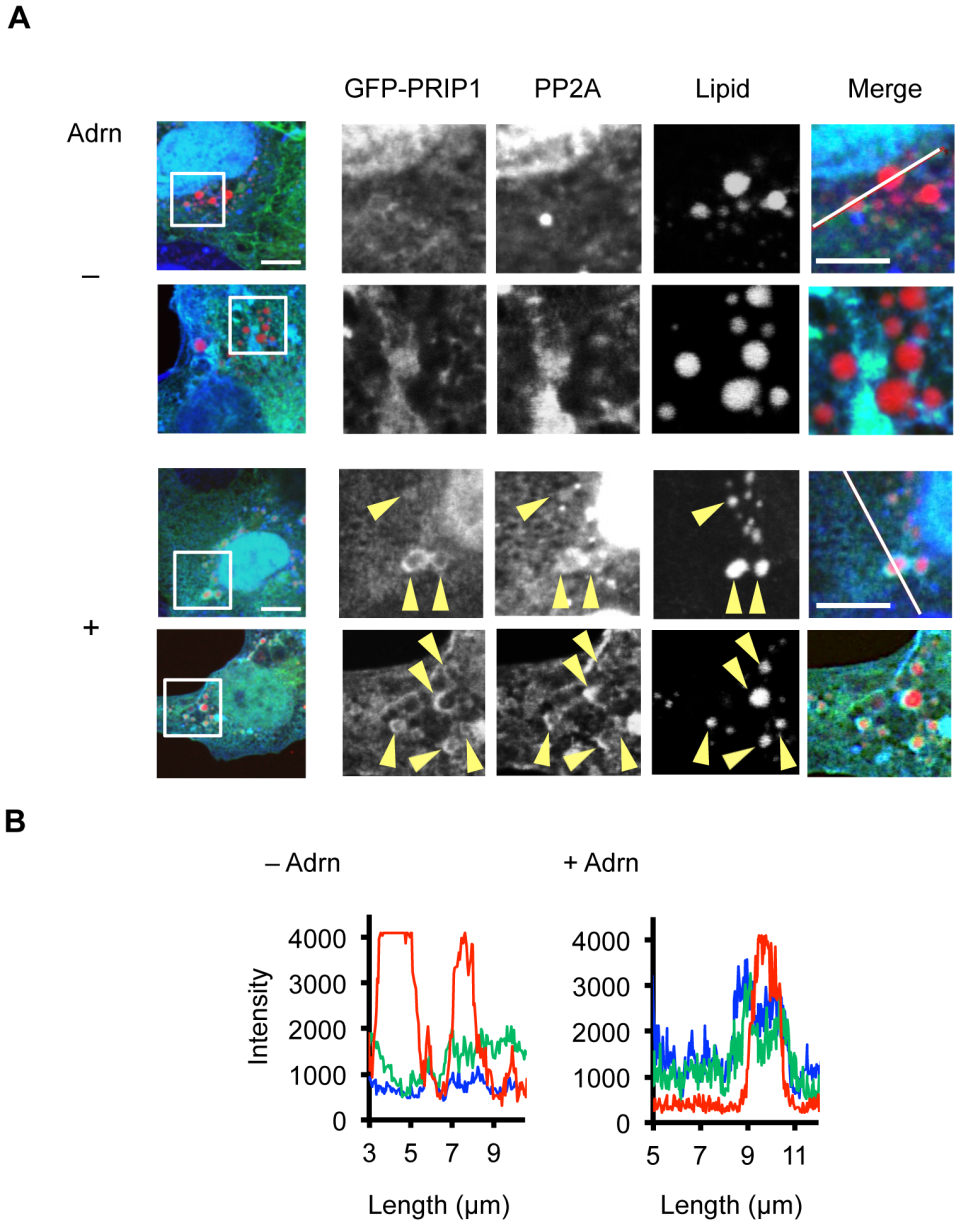
PRIP and PP2A are translocated to the periphery of lipid droplets in COS7 cells after adrenaline stimulation. (A) COS7 cells cultured with oleic acid with (+) or without (–) adrenaline stimulation (5 µM). Two sets of representative images are shown. The boxed areas of the left image (scale bar: 10 µm) are enlarged in the four right images (scale bar: 5 µm). Five similar images were obtained from three independent experiments. Arrowhead in GFP-PRIP1 and PP2A images indicates an accumulation of each signal at lipid droplet periphery. (B) Intensity profiles along the white lines in the merged images of (A). Compared with unstimulated cells (–Adrn), adrenaline-stimulated cells (+Adrn) exhibit increased fluorescence intensity corresponding to GFP-PRIP1 (green) and PP2A (blue) at the edge of the lipid droplet (red).

To observe the translocation of PRIP, PP2A, and PP1 to lipid droplets, we performed immunocytochemical analysis using COS7 cells. COS7 cells transfected with GFP-fused PRIP1 were cultured overnight with oleic acid, which is commonly used to induce lipid droplet formation in cultured cells, and then stimulated with 5 µM adrenaline for 5 min. After fixation, PP2A and PP1 were visualized with their specific antibodies. As shown in Fig. 5A, GFP-PRIP1 and PP2A signals in the cytosol were diffuse under basal conditions. However, after adrenaline stimulation, both GFP-PRIP1 and PP2A signals accumulated at the peripheries of lipid droplets (Fig. 5A, B). Translocation of PP1 to lipid droplets could not be observed clearly (data not shown), probably because of the abundance in the cells. Similar observations were made with PRIP2 (Fig. S2). Together, these results indicate that adrenaline stimulation induces the translocation of PRIP1 and PRIP2 from the cytosol to the lipid droplet membrane along with PP2A and possibly PP1.

### PRIP-mediated protein phosphatase activity is important for HSL dephosphorylation

**Figure 6:**
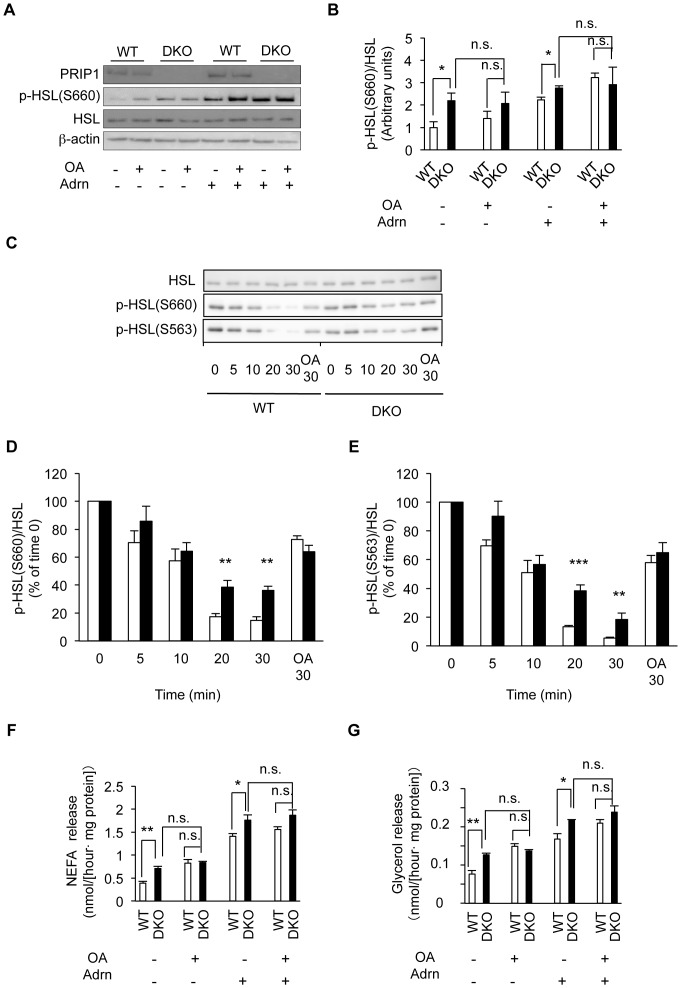
PRIP-mediated protein phosphatase dephosphorylates HSL Ser660 and Ser563 and regulates the release of fatty acids and glycerol in adipose tissue. (A, B) Effect of protein phosphatase activity on HSL Ser660 phosphorylation after combination stimulation by adrenaline (Adrn) and OA. A set of typical images from three independent experiments is shown (A). (C–E) Analysis of the dephosphorylation process of HSL. White adipose explants were stimulated with 5 µM adrenaline for 30 min and then cultured with a β3-adrenergic receptor antagonist (SR59230A, 20 µM) and a PKA inhibitor (PKI 14–22, 10 µM) for the indicated times to monitor the HSL dephosphorylation state. The dephosphorylation assay was also performed in the presence of 1 µM OA for 30 min (lane: OA30). Samples were homogenized, and western blot analysis was conducted using anti-p-Ser660 and anti-p-Ser535 HSL antibodies. A typical image from three independent experiments is shown. WT, white bar; DKO, black bar in (D, E). (F, G) Measurement of NEFA (F) and glycerol (G) released from cultured adipose tissues after combination stimulation by Adrn and OA (n = 3 experiments). OA: 1 µM okadaic acid, Adrn: 1 µM adrenaline. The data represent mean ±SEM. *P<0.05, **P<0.01, and n.s. (not significant).

We next examined the involvement of protein phosphatase activity in HSL phosphoregulation, with or without PRIP. Adipose tissues obtained from *PRIP*-DKO and WT mice were stimulated using a combination of adrenaline (1 µM) and OA (1 µM), a protein phosphatase inhibitor, and then the phospho-status of HSL at Ser660 was assessed. Under basal conditions (*i.e.*, without OA and adrenaline), the phospho-Ser660 level in the *PRIP*-DKO tissue was 2.2-fold higher than that of the WT tissue. When OA was added, the phospho-Ser660 level increased 1.4-fold in the WT tissue but was not altered in the *PRIP*-DKO tissue, indicating that protein phosphatase activity sensitive to OA is implicated in the dephosphorylation of HSL at Ser660 and that the phosphatase responsible for this activity is probably active in the presence of PRIP (Fig. 6A, B). Adrenaline stimulation increased the phosphorylation of HSL in both WT and *PRIP*-DKO adipose tissues, but the phosphorylation of HSL in the *PRIP*-DKO tissue was significantly higher than that in the WT tissue. Stimulation of adrenaline together with OA increased HSL phosphorylation to similar levels in both WT and mutant tissues (Fig. 6A, B). These data strongly argue that dephosphorylation of HSL by protein phosphatases in lipid droplets is attenuated in *PRIP*-DKO adipocytes, suggesting the involvement of a PRIP/phosphatase complex in basal and hormonally stimulated TAG hydrolysis.

To assess the time course of HSL dephosphorylation, white adipose tissues were first stimulated with adrenaline and subsequently treated with a β3-adrenergic receptor antagonist and PKA inhibitor for various durations (Fig. 6C). The fat-cake fractions were then analyzed for the phospho-status of HSL by immunoblotting with anti-p-HSL Ser660 and anti-p-HSL Ser563. In the WT explants, dephosphorylation of HSL at Ser660 and Ser563 was observed to occur gradually, and the phosphoprotein bands faded at 30 min (Fig. 6C, D). However, the dephosphorylation process in the *PRIP*-DKO explants was slow; the Ser660 and Ser563 phosphorylation levels were decreased to approximately 40% (Ser660 at 20 min and 30 min), 40% (Ser563 at 20 min), and 20% (Ser563 at 30 min) compared to the initial levels, respectively, which were significantly higher than those in WT mice (Fig. 6C–E). Dephosphorylation in both genotypes was inhibited by the addition of 1 µM OA for 30 min (see OA30 in Fig. 6C–E).

Finally, we assayed the culture medium for NEFA and glycerol released from adipocytes (Fig. 6F, G). The profiles of these compounds were much the same as that for the phospho-level of HSL at Ser660 in all cases.

## Discussion

This study was initiated by the observation that *PRIP*-DKO mice had smaller epididymal fat pads than WT mice, indicating that PRIP deficiency caused either a downregulation of lipid synthesis or an upregulation of lipid hydrolysis, or both. In preliminary experiments by using conventional differentiation-inducing agents [41], adipocyte differentiation was inhibited both in *PRIP*-knockdown NIH3T3-L1 cells and in *PRIP*-DKO mouse embryonic fibroblasts. Therefore, the reduction in size of adipose tissues observed in *PRIP*-DKO mice may partly be the result of reduced levels of adipocyte differentiation. We are currently investigating on how PRIP participates in adipogenesis. *PRIP*-DKO mice showed higher plasma levels of NEFA and glycerol compared those in WT mice, suggesting enhanced lipolysis activity in *PRIP*-DKO mice. Therefore, this study focused on the role of PRIP in lipid hydrolysis catalyzed by three major lipases, ATGL, HSL, and MGL.

Expression levels of these lipases as well as other proteins related to lipid metabolism such as ATGL, HSL, MGL, CGI-58, perilipin A, and the β3-adrenergic receptor were not different between *PRIP*-DKO and WT mice, suggesting an alteration of lipolysis regulation in *PRIP*-DKO mice. PKA mediates the activation of HSL by phosphorylation and the activation of ATGL through the phosphorylation of perilipin, both of which can be dephosphorylated by PP1 and/or PP2A [4], [8], [12]. Since we previously reported that PRIP is a binding partner of PP1 and PP2A, regulating the activities [23], [25]–[27], we examined whether the dephosphorylation of HSL and perilipin A was regulated by PRIP.

In WT mice, starvation (*in vivo*) and adrenaline treatment (*ex vivo*) stimulated the translocation of PRIP1 and PRIP2 from the cytosol to lipid droplets. Likewise, in response to adrenaline stimulation, PP1 and PP2A were enriched in lipid droplets, thereby showing higher catalytic activities in WT but not in *PRIP*-DKO adipocytes. The subcellular distribution analysis revealed that in WT adipocytes, the amount of PP1 accumulation in lipid droplets in response to adrenaline stimulation was less than that of PP2A. In addition, accumulation of PP1 at the periphery of lipid droplets was almost undetectable in immunocytochemical experiments using COS7 cells cultured with oleic acid, although the accumulation of PP2A and PRIP at the periphery was observed. These data suggest that PP2A may play a larger role in the dephosphorylation process on the lipid droplet surface than PP1.

In adipocytes, lipolysis is triggered by the translocation of HSL to lipid droplets, followed by the catalytic activation of HSL by PKA-mediated phosphorylation at Ser563 and Ser660 [13], [14]. PP2A dephosphorylates HSL at Ser563 and Ser660 [8], [9]. Since PP2A bound to PRIP is catalytically active [26], [27], the accumulation of PP2A together with PRIP at the surface of lipid droplets promotes HSL dephosphorylation in WT adipocytes. As there is less PP2A at the surface of lipid droplets in *PRIP*-DKO adipocytes than in WT adipocytes, dephosphorylation of HSL is decreased, which increases the level of lipolytic activity. Therefore, the present study of *PRIP*-DKO mice indicates that *PRIP* deficiency produces increased phosphorylation (*i.e.*, activation) of HSL, leading to the increase of lipolysis products observed in *PRIP*-DKO mouse plasma and explant culture medium.

Perilipin A is minimally phosphorylated under basal conditions and forms a barrier on lipid droplets that shields TAG stored in the droplets from cytosolic lipases. However, fasting promotes the phosphorylation of perilipin A, which then facilitates the translocation of phosphorylated HSL to lipid droplets, where this potent lipase gains access to TAG and DAG substrates to catalyze lipolysis [39], [42], [43]. Furthermore, ATGL activity involved in the initial step of TAG hydrolysis, is also regulated by PKA-dependent phosphorylation of perilipin A [44]. Under basal condition, perilipin A sequesters CGI-58 to prevent ATGL activation. Starvation triggers the phosphorylation of perilipin A and thus releases CGI-58, resulting in the promotion of the association between CGI-58 and ATGL, and subsequent activation of ATGL. In this study, perilipin A in lipid droplets in *PRIP*-DKO adipocytes was highly phosphorylated under both fed and fasting conditions; therefore, the lipolytic activity might be higher in *PRIP*-DKO mice than in WT mice. PP1 predominantly dephosphorylates perilipin [7]; therefore, perilipin phosphorylation correlates with the activity of PP1 in lipid droplet fractions. In explant experiments, the amount and activity of PP1 in the *PRIP*-DKO lipid droplet fraction was significantly lower than that of the WT fraction. Under basal condition, the higher levels of phosphorylated perilipin A seen in *PRIP*-DKO adipocytes might be related to this observation. However, further experiments are apparently needed to understand the PP1-regulated perilipin-mediated lipolysis pathway.

After fasting for 5 h, *PRIP*-DKO mice exhibited approximately 18% lower serum glucose (91.4±1.3 mg/dL; n = 5) than WT mice (110.8±2.9 mg/dL; n = 5). This likely resulted from increased insulin secretion from pancreatic β-cells [45], [46]. Intracellular glucose is metabolized by glycolysis to produce acetyl-CoA, which is both an end product of glycolysis and an initial substrate for fatty-acid synthesis. Accordingly, lower levels of glucose yield lower levels of acetyl-CoA. Further studies are apparently required for analyzing the linkage of this feature to the whole metabolic phenotype of *PRIP*-DKO mice.

**Figure 7:**
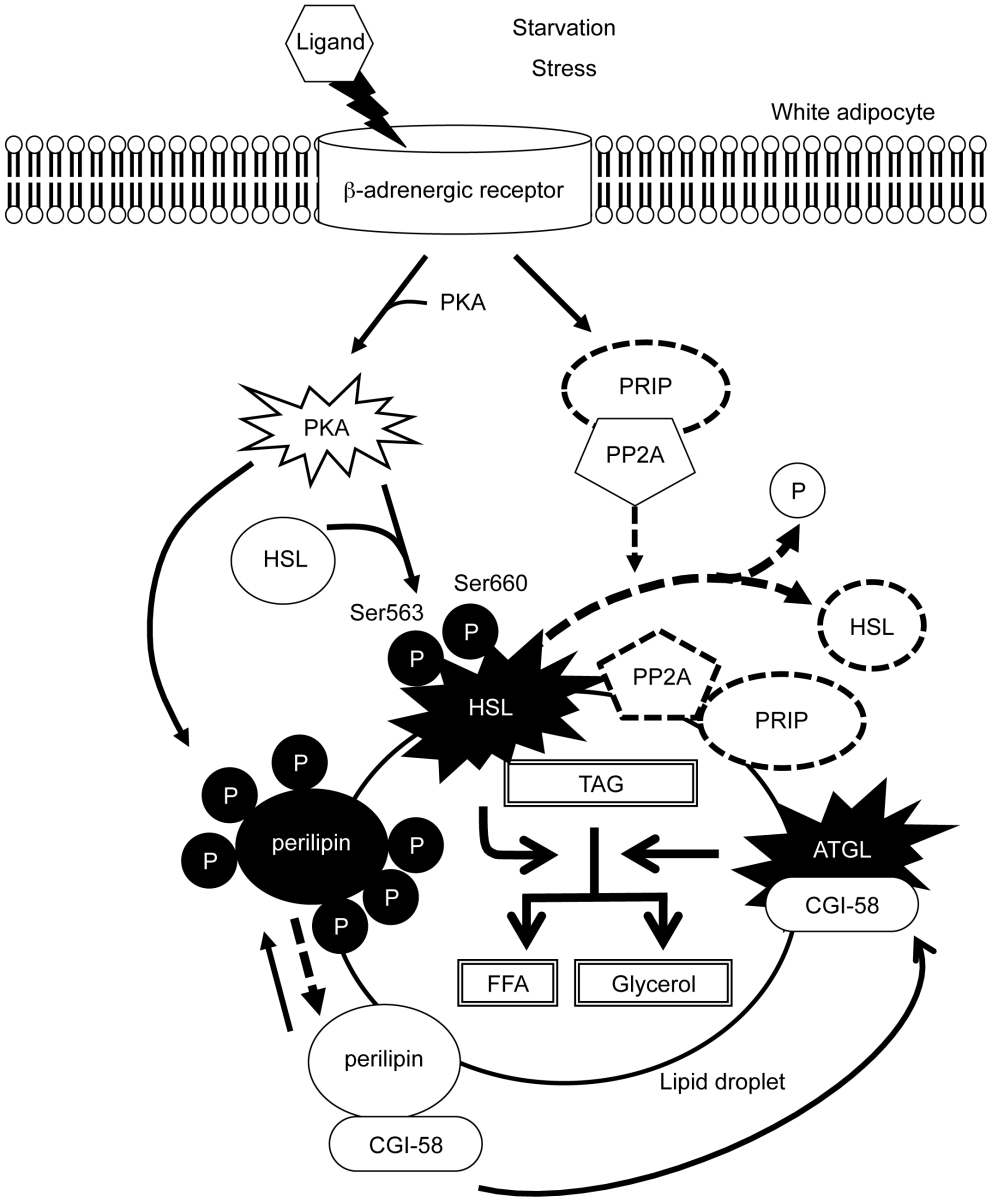
Possible mechanism by which the PRIP/protein-phosphatase complex mediates lipolysis. Lipolysis in adipocytes is mediated by the activation of a PKA-mediated pathway. The process is regulated by lipases (HSL and ATGL) and other modulatory proteins, including perilipin, CGI-58, PP2A, and PRIP (see Discussion). The disappearance of the dotted line represents postulated situations in *PRIP*-DKO mice. TAG, triacylglycerol; FFA, free fatty acid; P, phosphate group; PKA, protein kinase A; HSL, hormone-sensitive lipase; PRIP, phospholipase C-related catalytically inactive protein; PP2A, protein phosphatase 2A; CGI-58, comparative gene identification 58 (abhydrolase domain-containing protein 5).

The results from the present study help build a model of PRIP-mediated phospho-regulation of lipolysis, as depicted in Figure 7. Specifically, starvation triggers the activation of PKA, which phosphorylates HSL at Ser660 and Ser563, leading to the translocation of HSL to lipid droplets and the subsequent hydrolysis of lipids in adipocytes [13]. The signal also induces the translocation of PRIP and PP2A to lipid droplets, which promotes the dephosphorylation of HSL, and attenuates lipolysis. These sequential events yield a sharp transient activation of lipolysis to provide a fine-tuning of hormonal regulation. Inactivation process requires PRIP to effectively recruit PP1 and PP2A for dephosphorylation. Therefore, HSL phosphorylation (active state) would be long-lasting in the absence of PRIP. Indeed, we have previously reported the long-lasting phosphorylation of the neuronal β subunit of GABA_A_ receptors and exocytotic SNAP-25 in *PRIP-DKO* mice [26], [28].

Phosphorylation of perilipin releases CGI-58, resulting in the activation of ATGL by association with CGI-58. The events would also be regulated by phosphatases, whose activities are modulated by PRIP; therefore it is included in Figure 7. Since, in this study, we did not examined whether the association between perilipin and CGI-58 is regulated by PRIP, further studies are apparently needed to elucidate the dissociation regulated by PRIP and phosphatases (possibly PP1). In addition, PDE 3 is known to be up-regulated by the phosphorylation catalyzed by Akt [47]. PRIP might be involved in the process, as PRIP associates with phosphorylated (active form) Akt [27]. It is possible that PRIP triggers activation of PDE 3 or 4 to reduce cAMP concentration to stop the activation of PKA (this events is not depicted in Figure 7).

Despite evidence that protein phosphatases are involved in the lipolytic pathway, the associated regulatory mechanisms remain undefined [2]. In this study, we found that PRIP is a novel key modulator regulating lipolysis in adipocytes as shown in Fig. 7. Understanding of this regulatory pathway of lipolysis will provide an important insight into the treatment and control of obesity and lead to the development of new drugs.

## Supporting Information

Figure S1 **Subcellular localization of HSL and perilipin.** Homogenates of epididymal fat pads obtained from non-fasting mice were fractionated by centrifugation into three fractions: a floating fat-cake fraction (fat), a supernatant fraction (sup), and a pelleted membrane fraction (ppt). The fractions were subjected to immunoblotting using indicated antibodies.
(TIF)

Figure S2 **Accumulation of PRIP2 and PP2A at the peripheries of lipid droplets in COS7 cells after adrenaline stimulation.** COS7 cells were transfected with GFP-tagged PRIP2 and myc-tagged PP2A. The cells were cultured overnight with oleic acid and then stimulated with (+) or without (–) adrenaline (5 µM) for 5 min. PP2A was detected with an anti-PP2A antibody followed by anti-mouse IgG labeled with Alexa Fluor 405. The cells were visualized by confocal microscopy. Two sets of representative images are shown. Boxed areas of the left image (scale bar: 10 µm) are enlarged in the four right images (scale bar: 5 µm). Two independent experiments were performed, and similar images were obtained. The arrowhead in the GFP-PRIP2 and PP2A images indicates an accumulation of each signal at the lipid-droplet periphery.
(TIF)
