# Supplementary figures and images for: Phospholipase C-Related Catalytically Inactive Protein (PRIP) Regulates Lipolysis in Adipose Tissue by Modulating the Phosphorylation of Hormone-Sensitive Lipase
Source: PLoS One. 2014 Jun 19;9(6):e100559. doi: 10.1371/journal.pone.0100559 (PMC4064000; doi:10.1371/journal.pone.0100559)

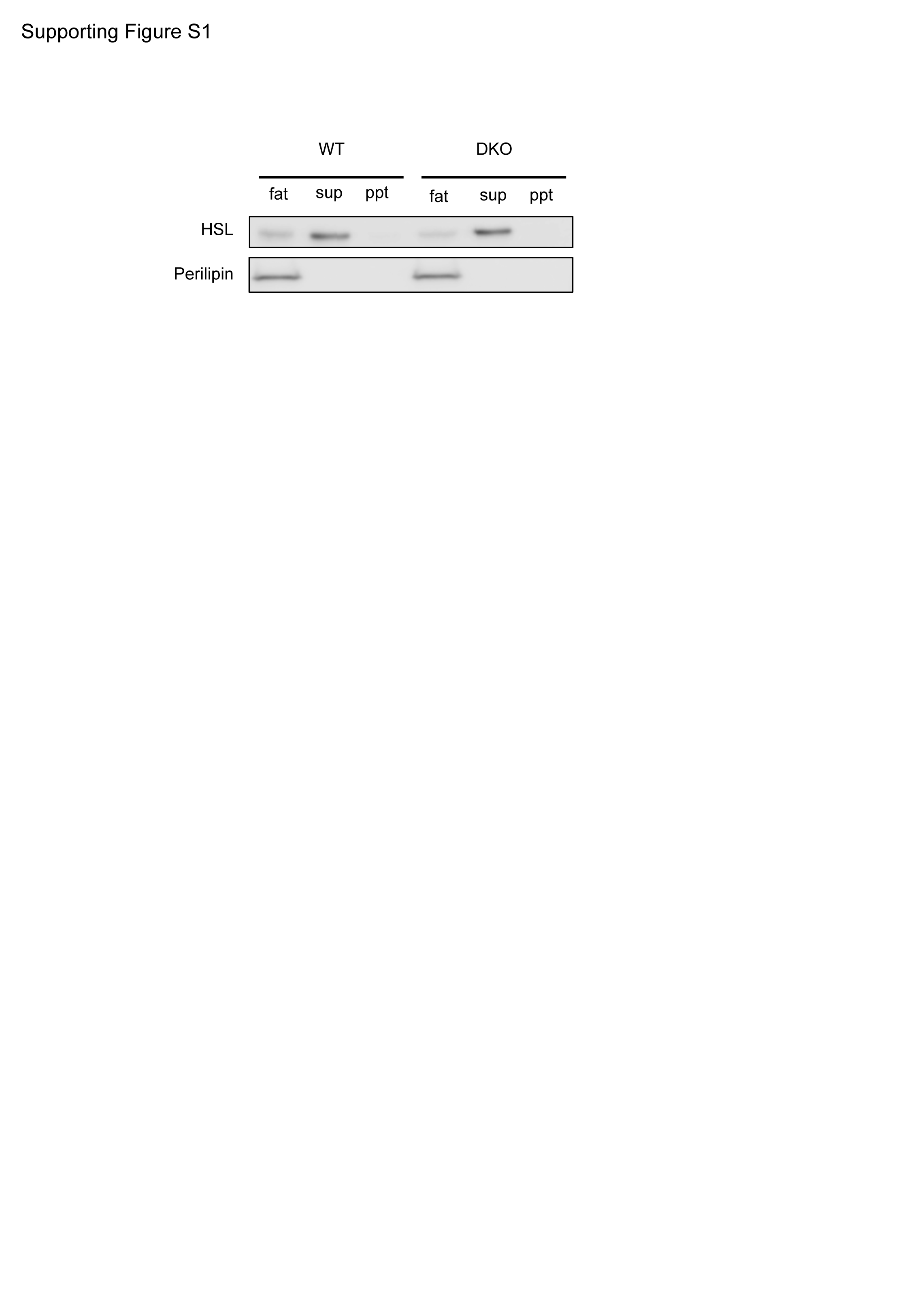

Supplement: Figure S1 — Subcellular localization of HSL and perilipin. Homogenates of epididymal fat pads obtained from non-fasting mice were fractionated by centrifugation into three fractions: a floating fat-cake fraction (fat), a supernatant fraction (sup), and a pelleted membrane fraction (ppt). The fractions were subjected to immunoblotting using indicated antibodies. (TIF) [file pone.0100559.s001.tif]

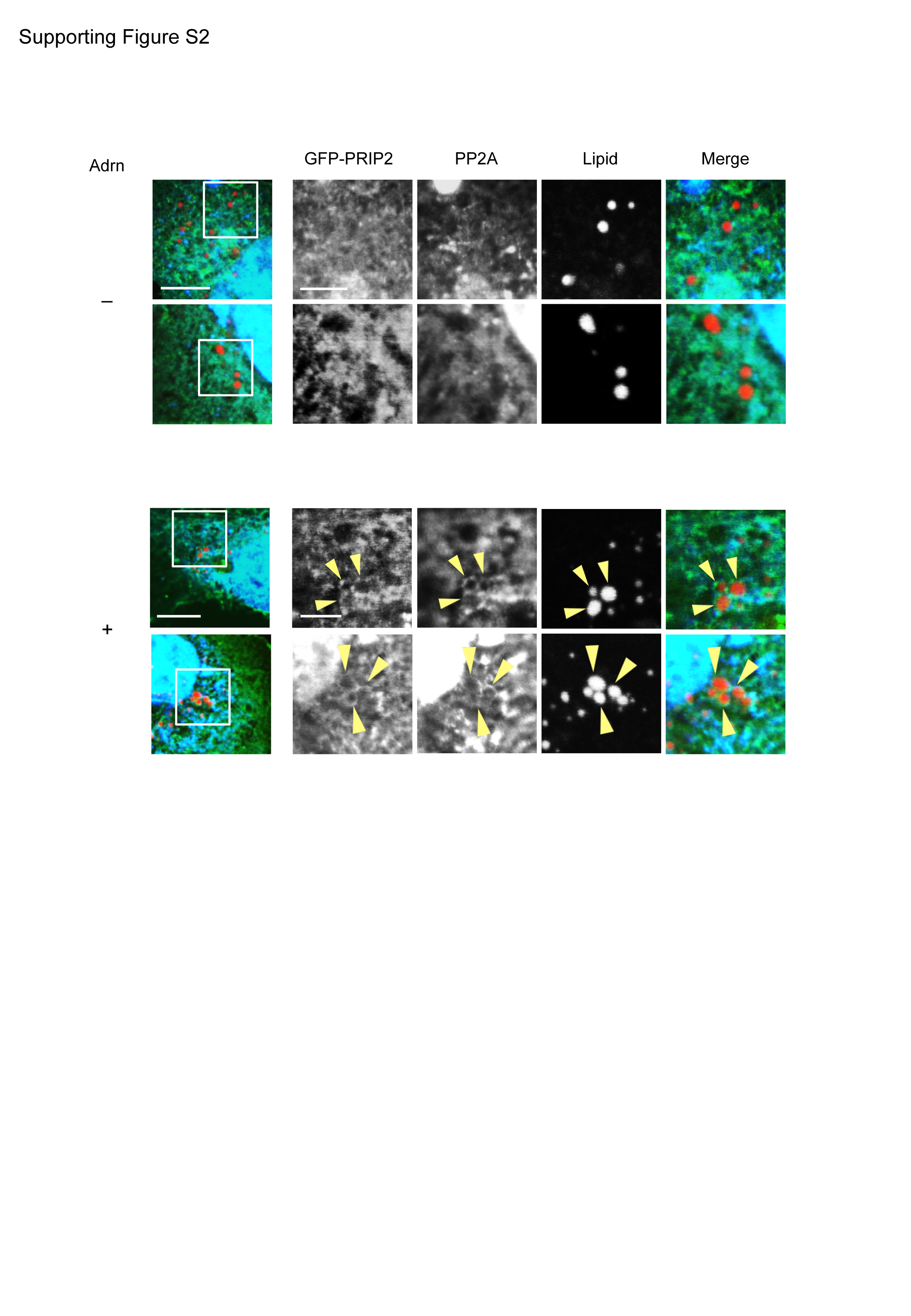

Supplement: Figure S2 — Accumulation of PRIP2 and PP2A at the peripheries of lipid droplets in COS7 cells after adrenaline stimulation. COS7 cells were transfected with GFP-tagged PRIP2 and myc-tagged PP2A. The cells were cultured overnight with oleic acid and then stimulated with (+) or without (–) adrenaline (5 µM) for 5 min. PP2A was detected with an anti-PP2A antibody followed by anti-mouse IgG labeled with Alexa Fluor 405. The cells were visualized by confocal microscopy. Two sets of representative images are shown. Boxed areas of the left image (scale bar: 10 µm) are enlarged in the four right images (scale bar: 5 µm). Two independent experiments were performed, and similar images were obtained. The arrowhead in the GFP-PRIP2 and PP2A images indicates an accumulation of each signal at the lipid-droplet periphery. (TIF) [file pone.0100559.s002.tif]
